# Psychometric properties of the incontinence utility index among patients with idiopathic overactive bladder: data from two multicenter, double-blind, randomized, Phase 3, placebo-controlled clinical trials

**DOI:** 10.1186/s12955-015-0306-5

**Published:** 2015-08-01

**Authors:** Nacho Castejón, Kristin Khalaf, Quanhong Ni, Jesús Cuervo, Donald L Patrick

**Affiliations:** LA-SER Outcomes, C/ Azcárraga 12 A 33010 Oviedo, Asturias, Spain; Allergan Inc., Irvine, CA USA; Allergan Inc., Bridgewater, NJ USA; Department of Health Services, University of Washington, Seattle, WA USA

**Keywords:** Overactive bladder, Urinary incontinence, Utility assessment, Health-related quality of life, I-QOL

## Abstract

**Background:**

Overactive bladder is a prevalent and burdensome condition. Generic utility measures may fail to reflect its full impact on patients’ health status. The Incontinence Utility Index (IUI) is a community-based preference index derived from the Incontinence Quality of Life Questionnaire (I-QOL) developed to value health states related to urinary symptoms in patients with neurogenic detrusor overactivity. This study assessed the measurement properties of the IUI in patients with idiopathic overactive bladder (OAB).

**Methods:**

Data were used from two clinical trials which recruited patients with OAB whose symptoms were inadequately managed with ≥1 anticholinergic medication. Psychometric evaluation included: Differential Item Functioning (DIF) analysis, concordance between I-QOL and IUI (Intraclass correlation coefficient [ICC], criterion and convergent validity according to relevant patient reported outcomes and clinical variables (Spearman’s correlation coefficient, rho), responsiveness, and agreement between utility measures (ICC and Bland-Altman method).

**Results:**

A total of 1,105 idiopathic OAB patients were included. Mean age (range) was 60.4 years (18–90), 87.8 % (*n* = 970) were female. DIF was identified in 3 items, none of which are contained in the IUI. ICC (CI95 %) was 0.944 (0.936–0.950). Statistically significant differences (p < 0.001) were found in IUI scores for patients improving according to the Treatment Benefit Scale (TBS). Moderate to strong correlations (rho > |0.6|) were found in the expected direction with daily incontinence, urgency episodes and disease-specific domains of King’s Health Questionnaire (KHQ). Low to moderate correlations (rho:<|0.6|) were found with Short Form version 2 (SF-12v2) summary components. A large effect size was found for patients reporting improvement (0.98–1.21) or great improvement (1.87–2.56) in the TBS, as well as in patients responding to treatment (1.19–2.40). Across utility measures, directional trends were consistent with OAB symptom profile, however, a lack of agreement in absolute values was observed.

**Conclusions:**

The IUI presents good psychometric properties for valuing the impact of UI-related problems in idiopathic OAB patients.

**Trial registration:**

ClinicalTrials.gov: NCT00910845 and NCT00910520.

## Background

Overactive bladder (OAB) is defined by the International Continence Society as “urgency, with or without urge incontinence, usually with frequency and nocturia” [1]. Prevalence rates of OAB range from 12 to 20 % in men and women and increase with age in both populations [2–5]. Previous research has shown that OAB, especially when accompanied with urinary incontinence (UI) or urgency urinary incontinence (UUI), considerably impacts patients’ health related quality of life (HRQoL) [6–9], affecting domains related to emotional well-being and social participation (e.g., avoidance and limiting behaviours) [7, 8], and also resulting in decreased work productivity [7, 10, 11].

Preference-based values, or utilities, are commonly used in health economics to weight health states associated with different conditions. These outcomes are of importance for economic resources allocation because they allow for estimation of Quality-Adjusted Life-Years (QALYs) by multiplying life expectancy with the utility value associated with a given health state. The QALY is considered an appropriate measure of health benefit because it reflects both mortality and health-related quality of life (HRQoL) impacts associated with a health condition [12]. Among the available approaches to obtain utilities, the administration of currently existing generic preference-based instruments such as the EuroQol-5D [EQ-5D]) [13–15] is commonly preferred by different health technology agencies in favor of promoting consistency and equity in decision-making and making this process more practical [12, 16–18]. However, in cases where generic utility instruments may not capture clinically important changes, as has been shown among patients with UI related problems [19, 20], disease-specific community-based utility instruments may be considered more appropriate.

The Incontinence Utility Index (IUI) was recently created for measuring the health states related to urinary symptoms in neurogenic patients with OAB and UI symptoms [21]. This tool was derived from the previously validated I-QOL questionnaire and its neurogenic module [22, 23] in accordance with the latest methodological recommendations [24]. The I-QOL is a widely used instrument in clinical research to address patients’ HRQoL associated with experiencing urinary problems [8, 22, 25, 26]. Development of the IUI was comprised of two stages and has been described in detail previously [21].

The IUI was originally developed from a sample of patients with neurogenic detrusor overactivity (NDO) and UI related symptoms, hence new research is needed to support use of this instrument in other populations experiencing lower urinary tract symptoms. Consequently, the present study was conducted to test the measurement properties of the IUI among patients with idiopathic OAB. The performance of the abbreviated health state classification system underlying the IUI and the utility estimates resulting from its application were compared with the original I-QOL and two other utility measures collected from the same sample, respectively.

## Methods

### Sample

OAB cases included in the present analysis were adult patients with idiopathic OAB and UI, participating in two multicenter, international, Phase 3, randomized, double-blind, placebo-controlled studies to evaluate the safety and efficacy of a single treatment of BOTOX® (onabotulinumtoxinA, Allergan Inc.). Eligible patients had idiopathic OAB with UI, were considered to be inadequately managed by anticholinergic therapy (insufficient efficacy or intolerable side effects), and experienced ≥3 episodes of urinary urgency incontinence (UUI) in a 3-day patient bladder diary, an average of ≥ 8 micturitions per day and a post-void residual urine volume ≤100 ml. A full description of their characteristics and study design can be found elsewhere [27, 28].

In order to test the differential item functioning by sample etiology, the sample originally used for item selection of the IUI is also included in this study. This sample was comprised of patients with UI due to NDO as a result of spinal cord injury or multiple sclerosis, recruited during two multicenter, double-blind, randomized, placebo-controlled, parallel-group studies [29, 30].

### Ethics, consent and permissions

Before entering in the aforementioned clinical trials all patients had to provide their written informed consent and all the studies were conducted in full compliance with the ethical principles regarding human experimentation of the Declaration of Helsinki. The New York University School of Medicine IRB, with Federal Wide Assurance number 00004952 reviewed the studies.

### Clinical variables and outcomes measures

Basic clinical and socio-demographic data were collected to describe the sample of the study. The following patient reported outcomes (PROs) were administered to patients in these studies:

- Treatment Benefit Scale (TBS) [31]: a single-item measure that evaluates patients’ perception of benefit following treatment. Responses are defined as 1 = greatly improved; 2 = improved; 3 = not changed; 4 = worsened. The TBS has demonstrated validity and responsiveness in previous clinical trials with antimuscarinic treatments in patients with OAB [31].
- Short Form-12 Health Survey version 2 (SF-12) [14, 32, 33]: A generic HRQoL questionnaire that includes 12 items from the SF-36 Health Survey [34] and has two component summary scores (physical –PCS- and mental –MCS-). Scores from patients’ responses are normalized to a distribution of 50 ± 10 (using U.S. general population norms), and a higher score indicates better HRQoL. It has demonstrated adequate psychometric properties to estimate the health burden of chronic conditions in general population surveys [33, 35]. A preference-based weighted index can be estimated from this instrument (SF-6D) following the models proposed by Brazier et al. [14]. The range of the observed utility values varies between 0.30 (worst health state) to 1.0 (best health state) [36].
- King’s Health Questionnaire (KHQ) [37, 38]: This self-administered disease-specific HRQoL questionnaire for UI has 21 items (4-point Likert scale) covering 8 domains: urinary symptom severity, role limitations, physical functioning, social functioning, emotional problems, personal relationships, sleep disturbance, and general health. The range of scores for each domain is between 0 and 100, with higher scores indicating greater impact on patients’ HRQoL (worse perceived health status). A condition-specific preference-based index has been developed from this instrument through reducing the number of dimensions (from 8 to 5), and valuing the resulting health state classification framework using direct elicitation (standard gamble) in a representative sample of patients with UI attending UK hospital outpatients clinics. Different models were tested to better adjust the predicting valuations for all the possible health states defined by this utility measure (*n* = 1024) [38]. Mean utility values obtained with the KHQ range from 0.77 to 0.98.
- Incontinence Quality of Life Questionnaire (I-QOL) [22, 23, 26]: This self-administered questionnaire comprises 22 items (5 Likert point) distributed into three dimensions. These principal domains are: avoidance and limiting behavior (items 1–4, 10, 11, 13 and 20), psychosocial impact (items 5–7, 9, 15–17, 21 and 22), and social embarrassment (items 8, 12, 14, 18 and 19). A total scale score is calculated by summing the scores of all items included in an scale and transforming them into a 0–100 scale (higher scores reflect better HRQoL) [22].

**Fig. 1:**
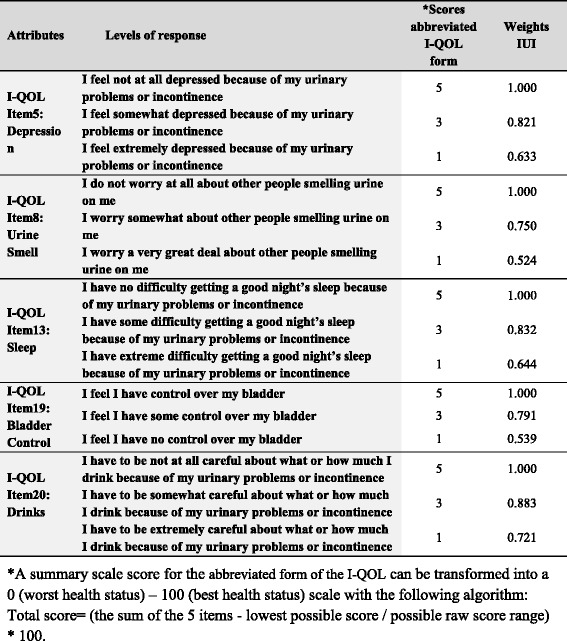
Incontinence Utility Index (IUI) attributes and levels

The abbreviated health states classification system comprises 5 items or attributes (Fig. 1). Utility scores can be derived from this instrument by applying the IUI algorithm: IUI utility score = 1.051 (b1 * b2 * b3 * b4 * b5)—0.051, where b is the estimated weight attached to the 3 different levels of each of the 5 attributes. The IUI has a utility score ranging from 0.036 (worst health state) to 1 (perfect health) [21].

### Statistical approach

A number of analyses were undertaken to assess the performance of the IUI in this sample of OAB patients:

Rasch analysis was used to check for the presence of differential item functioning (DIF, or measurement bias) of the I-QOL between patients with OAB and NDO patients from which the IUI was originally developed. Items were calibrated and subjects were scored using the Partial Credit Model [39]. DIF was tested by segmenting the sample by etiology [40]. The presence of DIF suggests that patients with the same disease severity tend to respond differently to an item depending on their disease etiology. DIF was considered significant with p < 0.001, and relevant if the difference between groups exceeded 0.5 logits.

In addition, further tests were applied to assess the psychometric performance of the abbreviated health state classification defined in the IUI and the associated utility scores in this sample of OAB patients.

### Concordance between I-QOL versions

The agreement between the original I-QOL and its abbreviated health state classification system in OAB patients was analyzed by applying the intraclass correlation coefficient (ICC) at baseline and at week 12. Two-way mixed effects models (people effects are random and measures effects are fixed) was applied with an absolute agreement type with average measures (reliability of the mean of the instruments). It is generally recommended that ICC be at least 0.7 [41], with higher values indicating better concordance.

### Validity

Criterion validity was assessed by studying the differences in both the I-QOL and the abbreviated form according to TBS scores at Week 12. Kruskal-Wallis (with Bonferroni’s correction for multiple comparisons) tests were conducted for this purpose. Statistically significant differences between TBS levels (p < 0.05) were expected in the scores of the I-QOL and its derived measures. Next, the relationship between the I-QOL, its abbreviated health state classification system, and the IUI with other PRO and clinical variables were evaluated. Spearman rank correlations coefficients (rho) were calculated between these instruments and the following clinical variables: age, volume voided per micturition (mL/24 h), daily incontinence episodes, daily urgency episodes, daily micturition episodes, daily incontinence urgency episodes, number of daily nocturia episodes and weekly incontinence episodes. Finally, convergent validity of the I-QOL, the abbreviated health state classification system, and the IUI were studied by testing its association (rho) with respect to the KHQ global score and domains, the SF-12 (Physical and Mental Component Summaries) and the utility scores derived from the KHQ and the SF-12 using Spearman rank correlation. A moderate to strong association (rho ≥0.5) was expected between the I-QOL and other disease-specific variables (e.g., disease-specific domains of the KHQ or the number of incontinence episodes), while the association between the I-QOL and variables less directly related to OAB (e.g., generic domains of the KHQ and the SF-12 summary components) was hypothesized to be lower (rho between 0.3 and 0.49).

### Responsiveness

The ability of the abbreviated form of the I-QOL and the IUI to capture clinically relevant changes in OAB patients were analyzed according to the level of response to treatment and TBS scale. To this end, differences in scores between baseline and Week 12 visits were calculated via Wilcoxon tests, standardized response means (SRMs) and effect size statistics. Patients were classified as respondents according to clinical criteria depending on the average percentage reduction in daily UI episodes from baseline: 50 %, 75 % or 100 % of reduction in daily episodes according to the 3-day bladder diary at week 12 [42]. SRMs were calculated as the mean change score (score at week12 minus score at baseline) divided by the standard deviation of change score. Effect size statistics were calculated as the mean change score divided by the standard deviation at baseline [8]. It was hypothesized that higher effect sizes were to be found between patients with a higher response or perceived benefit at week 12. Conventional benchmarks to interpret effect sizes are as follows: an effect size of 0.2–0.49 is considered small, 0.5–0.79 medium and over 0.8, large [43].

### Agreement between utility measures

Bland-Altman diagrams for agreement and the ICC statistic (with a two-way mixed effects model and checking an absolute agreement type with average measures) at baseline and Week 12 were used to study to what extent the utility values from the IUI and those obtained from the SF-12 [14] and the KHQ [38] could be interchangeable. The limits of agreement in the Bland-Altman figures were defined at a distance from the mean of 1.96 times the standard deviation of the differences. Acceptable limits of concordance of ± 0.1 points from 0 (maximum concordance in mean values) are also displayed to represent relevant discrepancies in common utility scales [44, 45].

The statistical packages SPSS 21.0 and Winsteps 3.75 were used to conduct the analyses above.

## Results

A total of 1,105 idiopathic OAB patients with UI symptoms were included. A majority of them were female (87.8 %) and Caucasian (90.9 %). Mean age (range) was 60.4 years (18 to 90). Patients had a median duration of OAB of 5 years and reported a mean (SD) of 5.44 (3.62) daily UI episodes, 4.90 (3.43) daily urgency urinary incontinence episodes, 11.74 (3.78) daily micturitions, 8.57 (4.40) daily urgency episodes, and 2.11 (1.42) nocturia episodes per day at study baseline. A summary of select patient demographic and clinical characteristics can be found in Table 1.

**Table 1:** Select baseline demographic, clinical, and patient-reported characteristics: OAB pooled study population

Variables	Baseline		
	n	Mean	SD
Age	1105	60.36	13.90
Female (n, %)	970	87.8 %	
Caucasian (n, %)	1004	90.90 %	
Duration of OAB (years)	1104	6.09	7.07
Volume Voided Per Micturition (mL/24 h)	1021	153.60	62.57
Daily Micturition Episodes	1026	11.74	3.78
Daily Incontinence Episodes	1026	5.44	3.62
Daily Urgency Episodes	1026	8.57	4.40
Daily Urinary Urgency Incontinence Episodes	1026	4.90	3.43
Daily Nocturia Episodes	1026	2.11	1.42
I-QOL Total Summary Score	1101	34.42	18.77
I-QOL Avoidance and Limiting Behavior Score	1103	31,43	16.99
I-QOL Psychosocial Impacts Score	1103	42.62	24.29
I-QOL Social Embarrassment Score	1103	24.69	20.93
I-QOL Abbreviated Form	1100	36.12	18.31
IUI Scores	1100	0.22	0.14
KHQ Domains			
General Health Perceptions Score	1099	31.76	23.82
Incontinence Impact Score	1099	83.35	23.91
Role Limitations Score	1099	63.32	29.04
Social Limitations Score	1099	43.59	30.97
Personal Relationships Score	834	37.03	35.70
Emotions Score	1099	55.67	30.30
Sleep/Energy Score	1099	65.00	26.77
Severity Measures Score	1099	65.12	22.97
King’s Health Questionnaire (KHQ) Utility Score	1099	0.93	0.02
SF-12v2 Outcomes			
Physical Component Summary Score (PCS)	1096	43.22	10.03
Mental Component Summary Score (MCS)	1096	43.19	11.98
SF-12v2 Health Survey Utility Score (SF-6D)	1096	0.66	0.13

Rasch analysis showed three items presented statistically significant differential item functioning with a relevant effect with respect to etiology of urinary symptoms (neurogenic vs. idiopathic). Details can be found on Table 2. Items exhibiting DIF included content related to trips to the toilet, aging and sexual activity. However, as none of the affected items were included in development of attributes and subsequent preference elicitation for the IUI no further action was deemed necessary.

**Table 2:** Rasch analysis: differential item functioning by etiology (NDO vs. OAB)

Item		NDO measure	NDO S.E.	OAB measure	OAB S.E.	DIF contrast	Welch t	p-value
1	I worry about getting to toilet in time	0.30	0.04	0.65	0.04	−0.35	−6.03	<0.001
2	I worry about coughing and sneezing	−1.17	0.04	−0.85	0.03	−0.31	−6.50	<0.001
3	I’m careful when standing up	−0.95	0.04	−0.71	0.03	−0.24	−5.07	<0.001
4	I worry about where toilets are located	0.36	0.04	0.61	0.04	−0.24	−4.33	<0.001
5	I feel depressed due to urinary problem^a^	−0.35	0.04	−0.26	0.03	−0.08	−1.65	0.099
6	I don’t feel free to leave my home	−0.21	0.04	−0.35	0.03	0.14	2.94	0.003
7	I’m prevented from doing what I want	0.24	0.04	0.10	0.03	0.14	2.62	0.009
8	I worry about a urine smell on me^a^	−0.40	0.04	−0.33	0.03	−0.06	−1.33	0.184
9	Urine problems are always on my mind	0.50	0.05	0.50	0.04	0.00	0.00	1.000
10	I make frequent trips to the toilet	0.27	0.04	0.96	0.04	**−0.69**	−11.3	<0.001
11	Important to plan details in advance	0.30	0.05	−0.09	0.03	0.39	6.93	<0.001
12	I worry urine problems will worsen w/age	0.27	0.04	0.86	0.04	**−0.59**	−10.3	<0.001
13	I can’t get a good night of sleep^a^	−0.13	0.04	0.03	0.03	−0.17	−3.39	0.001
14	I worry about being embarrassed	0.41	0.04	0.39	0.03	0.02	0.42	0.674
15	I feel like I’m not a healthy person	−0.34	0.04	−0.56	0.03	0.22	4.45	<0.001
16	I feel helpless due to urinary problems	−0.52	0.04	−0.52	0.03	0.00	0.00	1.000
17	I get less enjoyment out of life	−0.16	0.04	−0.26	0.03	0.10	2.00	0.045
18	I worry about wetting myself	0.83	0.05	0.81	0.04	0.02	0.34	0.735
19	I feel I can’t control my bladder^a^	1.12	0.05	0.74	0.04	0.37	5.68	<0.001
20	I watch what and how much I drink^a^	0.51	0.05	0.36	0.03	0.15	2.61	0.009
21	My choice of clothing is limited	−0.53	0.04	−0.68	0.03	0.15	3.13	0.002
22	I worry about having sex	−0.55	0.03	−1.14	0.03	**0.59**	13.13	<0.001

DIF: differential item functioning ^**a**^Items which constitute the descriptive system of the IUI. DIF contrast in bold indicate items which exhibited differential item functioning

A high level of agreement was found between the abbreviated form of the I-QOL and the original version at both baseline and week 12: ICC = 0.900 (CI 95 %: 0.886–0.912) and 0.944 (CI 95 %: 0.936–0.950). A statistically significant difference was seen for I-QOL scores, the abbreviated health state descriptive system, and the IUI scores across perceived benefit levels after 12 weeks of treatment (TBS scale, Kruskal-Wallis Test p < 0.001, Table 3). Pairwise comparisons with Bonferroni correction were also statistically significant, with exception of scores between patients who indicated that their health condition had either not changed or worsened.

**Table 3:** Differences in the I-QOL and IUI scores according to the Treatment Benefit Scale (TBS) at week 12

	Treatment benefit scale	Number	Mean	SD	95 % CI for mean		p-value
					Lower	Upper	
I-QOL scale score	Greatly improve	187	77.49	20.70	74.51	80.48	<0.001
	Improved	274	57.36	22.69	54.67	60.06	
	Not changed^a^	464	37.21	20.42	35.35	39.08	
	Worsened^a^	109	31.46	19.95	27.67	35.25	
Scale score of the abbreviated form of the I-QOL	Greatly improve	187	72.19	20.77	69.20	75.19	<0.001
	Improved	274	54.71	19.97	52.33	57.08	
	Not changed^a^	464	37.95	19.23	36.20	39.71	
	Worsened^a^	109	33.58	18.64	30.04	37.12	
IUI scores	Greatly improve	187	0.60	0.27	0.56	0.64	<0.001
	Improved	274	0.39	0.20	0.37	0.42	
	Not changed^a^	464	0.24	0.16	0.23	0.25	
	Worsened^a^	109	0.21	0.14	0.18	0.23	

^**a**^After applying Bonferroni’s correction, all comparisons established by TBS levels were significant with the exception of that between not changed vs worsened levels

Table 4 shows the relationship between I-QOL versions, clinical variables and other HRQoL instruments. As hypothesized, correlation coefficients reflected a negative and moderate to strong association between I-QOL versions and those clinical variables related to UI problems (rho > |0.6|): daily incontinence episodes and urgency episodes, indicating that greater frequency of UI related problems was associated with lower I-QOL scores. Age was not significantly related to I-QOL scores. Furthermore, the scores obtained from the I-QOL and its derived instruments were negative and moderately to strongly associated with disease-specific HRQoL domains of the KHQ (rho > |0.6–0.8|), with the Severity Measures and Emotions domains having the strongest relationship to I-QOL measures. As expected, the association with the summary components of the SF-12, a measure of general HRQoL, were positive but low to moderate: low \with respect to the physical component summary score (rho range: 0.25–0.31) and moderate relative to the mental component summary score (rho range = 0.47–0.52).

**Table 4:** Responsiveness of I-QOL and IUI across clinical criteria at week 12

		Change score^a^				p-value	SRMs^b^	Effect size
	Number	Mean	SD	Lower limit	Upper limit			
TBS^c^ Greatly improve								
- Abbreviated I-QOL	186	34.62	22.78	31.33	37.92	<0.001	1.52	1.87
- Original I-QOL	187	42.79	21.72	39.66	45.92	<0.001	1.97	2.24
- IUI	186	0.37	0.27	0.33	0.41	<0.001	1.37	2.56
TBS Improved								
- Abbreviated I-QOL	274	18.14	19.64	15.76	20.40	<0.001	0.92	0.98
- Original I-QOL	274	22.80	19.82	20.40	25.08	<0.001	1.15	1.21
- IUI	274	0.16	0.19	0.14	0.19	<0.001	0.87	1.14
TBS Not changed								
- Abbreviated I-QOL	462	1.90	15.86	0.56	3.47	0.017	0.12	0.11
- Original I-QOL	462	2.20	13.84	0.98	3.51	0.004	0.16	0.12
- IUI	462	0.02	0.13	0.00	0.03	0.031	0.12	0.12
TBS Worsened								
- Abbreviated I-QOL	109	−0.73	15.97	−3.77	2.30	0.502	−0.05	−0.04
- Original I-QOL	109	−1.09	14.01	−3.75	1.57	0.376	−0.08	−0.06
- IUI	109	−0.01	0.13	−0.03	0.02	0.363	−0.05	−0.05
Responder^d^ 50 % = 0 (No)								
- Abbreviated I-QOL	552	2.79	16.86	1.49	4.36	<0.001	0.17	0.16
- Original I-QOL	552	2.82	14.89	1.65	4.19	<0.001	0.19	0.16
- IUI	552	0.02	0.14	0.01	0.03	<0.001	0.16	0.17
Responder 50 % = 1 (Yes)								
- Abbreviated I-QOL	470	22.47	23.09	20.34	24.49	<0.001	0.97	1.19
- Original I-QOL	471	28.51	24.01	26.29	30.60	<0.001	1.19	1.50
- IUI	470	0.23	0.25	0.20	0.25	<0.001	0.90	1.52
Responder 75 % = 0 (No)								
- Abbreviated I-QOL	697	5.05	17.89	3.77	6.46	<0.001	0.28	0.28
- Original I-QOL	697	5.85	17.29	4.61	7.20	<0.001	0.34	0.32
- IUI	697	0.04	0.15	0.03	0.05	<0.001	0.28	0.32
Responder 75 % = 1 (Yes)								
- Abbreviated I-QOL	325	26.40	23.65	23.85	28.94	<0.001	1.12	1.41
- Original I-QOL	326	33.46	23.76	30.86	35.96	<0.001	1.41	1.75
- IUI	325	0.28	0.27	0.25	0.31	<0.001	1.05	1.80
Responder 100 % = 0 (No)								
- Abbreviated I-QOL	843	7.38	19.10	6.12	8.72	<0.001	0.39	0.41
- Original I-QOL	843	9. 18	19.58	7.88	10.54	<0.001	0.47	0.50
- IUI	843	0.06	0.17	0.05	0.08	<0.001	0.38	0.48
Responder 100 % = 1 (Yes)								
- Abbreviated I-QOL	179	32.85	23.97	29.21	36.12	<0.001	1.37	1.79
- Original I-QOL	180	40.26	22.99	36.69	43.30	<0.001	1.75	2.10
- IUI	179	0.36	0.28	0.32	0.40	<0.001	1.29	2.40

^a^Change score = scores at week 12 minus scores at baseline

^b^SRM: Standardized response means

^c^TBS: Treatment Benefit Scale

^d^Responder: calculated from the average percentage of reduction in daily UI episodes from baseline

The responsiveness of I-QOL versions was evaluated using SRMs and effect sizes by week 12 TBS levels and responder definitions (Table 4). Scores from patients who reported an improvement in symptoms corresponded with a large effect size in all the I-QOL derived instruments (range: 0.98–1.21), with scores from patients reporting symptoms had “Greatly improved” showing higher effect sizes (range: 1.87–2.56). Furthermore, effect sizes were low between those patients reporting no changes or being worse at week 12 from baseline (range: 0.04–0.12). The same trend was observed in the case of the responder classification implemented according to the percentage of UI episodes reduction: the higher the reduction in the frequency of UI related symptoms, the higher the effect sizes. For instance, between patients reporting a benefit of 50 % of reduction in UI episodes, effect sizes ranged from 1.19 to 1.52 while among those with 100 % reduction the range was 1.79–2.40.

**Fig. 2:**
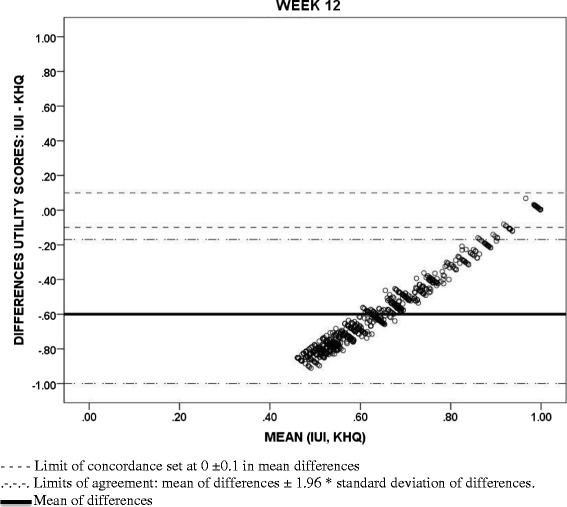
Bland-Altman scatterplots: Concordance between Incontinence Utility Index (IUI) and King’s Health Questionnaire (KHQ) utility scores

**Fig. 3:**
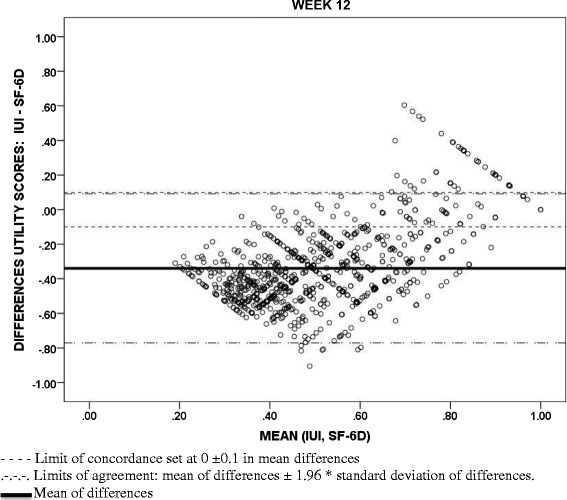
Bland-Altman scatterplots: Concordance between Incontinence Utility Index (IUI) and Short Form-12 Health Survey (SF-6D) utility scores

**Fig. 4:**
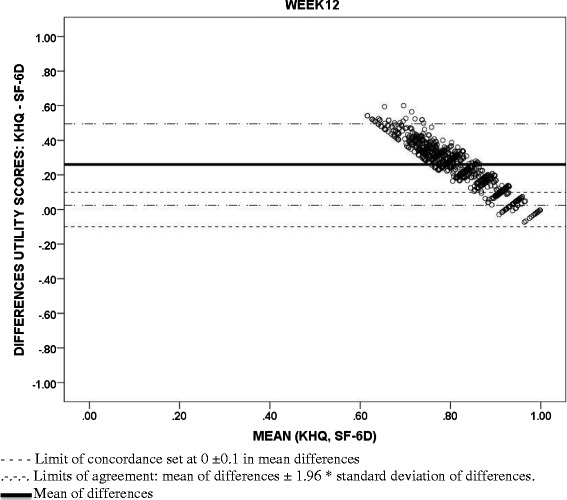
Bland-Altman scatterplots: Concordance between King’s Health Questionnaire (KHQ) and Short Form-12 Health Survey (SF-6D) utility scores

With regards to the concordance between utility values, although the associations between the IUI and the utilities scores from the KHQ and the SF-12 (SF-6D) were significant, positive and moderate to strong (0.761 and 0.469, respectively, p < 0.001) (Table 5), Bland-Altman methods highlighted poor agreement between these measures meaning that large differences in utility values were seen in the scatterplots (Figs. 2, 3 and 4). Mean differences (SD) between utility values from IUI and both KHQ and SF-6D were: −0.70 (0.13) and −0.44 (0.15) at baseline and −0.60 (0.22) and −0.34 (0.22) at week 12, respectively. The agreement between the utility scores obtained from the KHQ and the SF-6D was also low: mean differences of values (SD) was 0.27 (0.12) at baseline and 0.26 (0.12) at week 12, far exceeding acceptable concordance thresholds. This low agreement was confirmed by ICC results: all the comparisons (at baseline and at week 12) were under the accepted threshold (0.7) and ranged from 0.014 to 0.2.

**Table 5:** Association between I-QOL and IUI scores with clinical variables and measures at week 12

	I-QOL Scale score	Abbreviated form of the I-QOL Scale score	IUI Scores
Age in Years (*n* = 1038)	−0.038*	−0.026*	−0.032*
Volume Voided Per Micturition (*n* = 1021)	0.227**	0.236**	0.233**
Daily Incontinence Episodes (*n* = 1026)	−0.627**	−0.579**	−0.594**
Daily Micturition Episodes (*n* = 1026)	−0.375**	−0.359**	−0.347**
Daily Urgency Episodes (*n* = 1026)	−0.537**	−0.515**	−0.510**
Daily Incontinence Urgency Episodes (*n* = 1026)	−0.638**	−0.589**	−0.603**
Daily Nocturia Episodes (*n* = 1026)	−0.315**	−0.333**	−0.300**
Weekly Incontinence Episodes (*n* = 1025)	−0.626**	−0.579**	−0.594**
I-QOL: Avoidance and Limiting Behavior Score (*n* = 1038)	0.941**	0.853**	0.842**
I-QOL: Psychosocial Impacts Score (*n* = 1038)	0.964**	0.846**	0.841**
I-QOL: Social Embarrassment Score (*n* = 1038)	0.930**	0.873**	0.891**
KHQ Utility Score (*n* = 1038)	0.827**	0.775**	0.761**
General Health Perceptions Score (*n* = 1036)	−0.228**	−0.195**	−0.193**
Physical functioning (*n* = 1036)	−0.775**	−0.701**	−0.698**
Role Limitations Score (*n* = 1038)	−0.783**	−0.696**	−0.693**
Social Limitations Score (*n* = 1038)	−0.755**	−0.668**	−0.660**
Personal Relationships Score (*n* = 761)	−0.587**	−0.517**	−0.510**
Emotions Score (*n* = 1038)	−0.807**	−0.737**	−0.728**
Sleep/Energy Score (*n* = 1038)	−0.617**	−0.628**	−0.605**
Urinary symptoms severity score (*n* = 1038)	−0.805**	−0.763**	−0.773**
SF12 Utility Score (SF-6D) (*n* = 1034)	0.528**	0.476**	0.469**
Physical Component Summary Score (*n* = 1033)	0.309**	0.252**	0.254**
Mental Component Summary Score (*n* = 1033)	0.517**	0.477**	0.468**

Spearman correlation coefficient

*p > 0.05 |**p < 0.001

## Discussion

This study presents the measurement properties of the IUI in a sample of idiopathic OAB patients with UI symptoms who had not been adequately managed with anticholinergic therapy. The health states covered with this societal preference-based utility measure were originally extracted from the I-QOL and its neurogenic module using a large sample of adult patients with NDO [21]. The abbreviated health state classification version of the I-QOL containing the descriptive system of the IUI did not include any items from the neurogenic module, hence it was appropriate to test its psychometric performance in idiopathic OAB patients with UI to provide evidence for use in health economic analyses comparing interventions of interest within this patient population. In a recent study, the I-QOL was shown to be a robust HRQoL instrument for measuring the impact of urinary problems in similar sample of idiopathic OAB patients [8] so it was reasonable to further explore the validity of the IUI in this target population.

Results of the Rasch analysis showed that none of the items included in the abbreviated health state classification system derived from the I-QOL exhibited DIF; that is, the IUI assigned comparable scores to analogous health states in NDO and idiopathic OAB patients. Interestingly, the three I-QOL items which did exhibit DIF pertained to aspects of life which would be expected to differ between these two patient populations (i.e., trips to the toilet, aging, and sexual activity). NDO patients may have limitations in “Trips to the toilet” and “Sexual activity” independently of UI, and their perspective on aging may be different compared to those with OAB. Responses on the items whose attributes were ultimately included in the IUI did not systematically differ across each sample.

A high level of absolute agreement between the original I-QOL and the descriptive system of the IUI was demonstrated by the ICC, which showed a very high proportion of variability that was attributed to variation between patients. This high degree of concordance indicates that IUI scores are a reliable indicator of urinary symptom impacts as measured by the I-QOL. In addition, both the abbreviated version of the I-QOL and the IUI captured significant differences relative to clinically meaningful variables such as the reduction in the number of UI episodes or the treatment benefit perceived by patients as evaluated by the TBS [31]. Clinically meaningful outcomes relevant to urinary symptoms (daily number of UI episodes, UUI frequency and nocturia) were moderately or strongly associated with IUI scores. The large effect sizes (d > 0.8) found between baseline and week 12 IUI scores among patients with a reduction in the frequency of UI symptoms are in accordance to previous publications using the I-QOL in OAB patients [8, 22, 46].

Although all utility measures demonstrated consistent trends (e.g., lower utility values were associated with poorer urinary symptom profile across all utility measures), a low level of agreement in absolute values was systematically found between the utility values estimated from each of the instruments. IUI utilities had the lowest absolute values relative to both the KHQ (condition-specific) and the SF-6D (generic measure) at baseline and at week 12, while KHQ utility scores were the highest. Utility values were more reasonably similar only among patients whose health state was close to full or the most desirable health state. This lack of concordance and consistency across different utility measures to assess overall health status has been frequently described in the literature. For instance, this issue has been addressed when valuing the health states obtained in obese women with UI [47], or in musculoskeletal and cardiovascular diseases [48, 49] and in representative samples of the general population using generic instruments [50]. In the present study, these discrepancies between the IUI and the KHQ may be due to the different populations interviewed to derive the health state classification systems and to estimate the respective utility indexes. The health states described by the IUI were valued by the general population while those of the KHQ were assessed by UI patients attending hospital outpatient clinics [38]. In other pathologies, it has been highlighted that patients provided significantly different values to the health states related to the disease they experienced in comparison to general population [51, 52]. Given the existing heterogeneity, inclusion of more than one utility instrument should be considered in order to provide a more comprehensive evaluation of the possible range of the values attached to the health status of patients with certain health conditions [53].

A number of limitations in the present study should be addressed. First, although data for the analyses came from multiple global randomized phase III clinical trials, further research should be conducted to address region-specific nuances with respect to self-reported health status. The samples utilized in this study are comprised of a specific subset of patients with urologic problems, thus, inferences to other urology populations should be made with caution. Second, the majority of patients in the OAB studies (87.5 %) were female so additional testing is required before generalizing these conclusions to men with OAB. Despite these limitations, the present research demonstrates the IUI’s ability to detect clinically important differences in OAB patients with UI and differentiate well across different levels of reported treatment benefit and symptom improvement. Thus the psychometric performance of the IUI was comparable to the original I-QOL.

In conclusion, the IUI is a valid instrument for the assessment of patients with urological symptoms related to idiopathic OAB. The utilities obtained from its application represent the societal value of the health states described and offers additional insight to researchers and HTAs when assessing the benefits of urological interventions for OAB and UI problems.

## Abbreviations

BOTOX: OnabotulinumtoxinA
DIF: Differential item functioning
EQ-5D: EuroQol-5 dimension
HRQOL: Health-related quality of life
ICC: Intraclass correlation coefficient
I-QOL: Incontinence quality of life questionnaire
IUI: Incontinence utility index
KHQ: King’s health questionnaire
NDO: Neurogenic detrusor overactivity
OAB: Overactive bladder
PRO: Patient reported outcome
QALY: Quality-Adjusted Life-Years
SF-12v2: Short form 12 health survey version 2
SF-6D: Short form-6 dimension
SRM: Standardized response means
TBS: Treatment benefit scale
UI: Urinary incontinence
UUI: Urgency urinary incontinence
UK: United Kingdom
